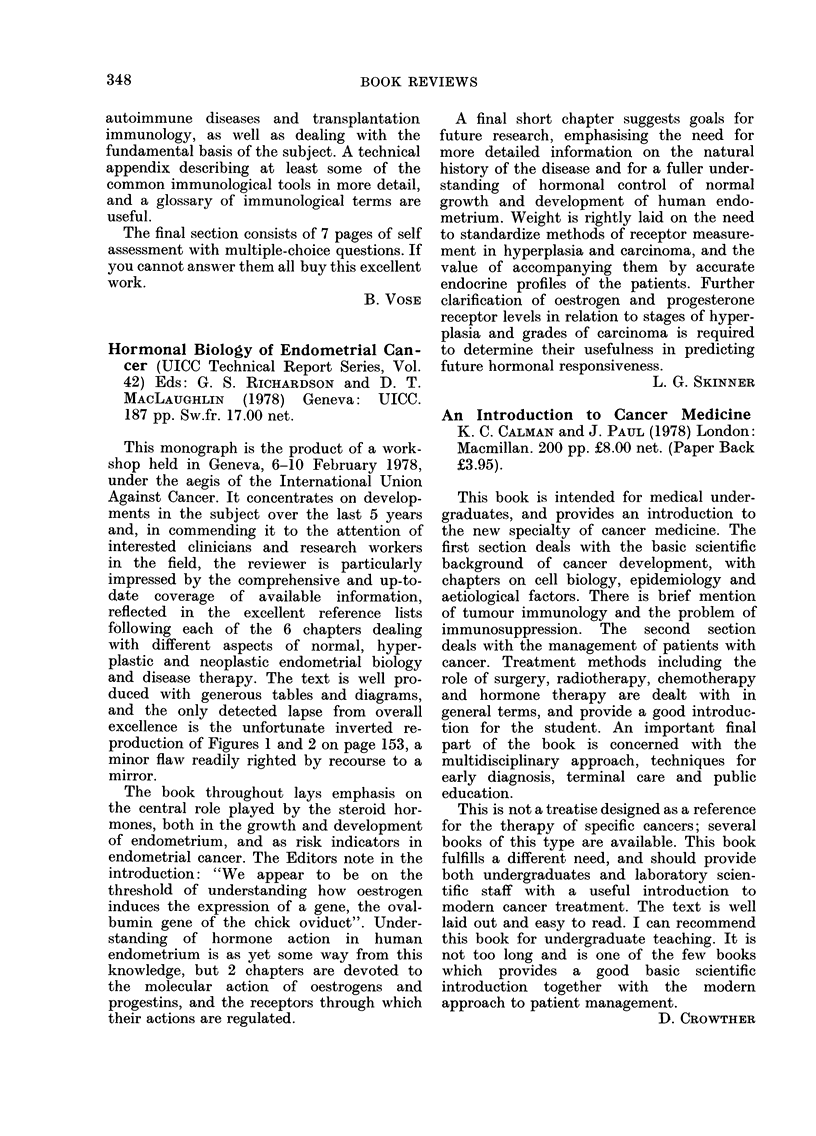# An Introduction to Cancer Medicine

**Published:** 1979-03

**Authors:** D. Crowther


**An Introduction to Cancer Medicine** K. C. CALMAN and J. PAUL (1978) London: Macmillan. 200 pp. £8.00 net. (Paper Back £3.95).

This book is intended for medical undergraduates, and provides an introduction to the new specialty of cancer medicine. The first section deals with the basic scientific background of cancer development, with chapters on cell biology, epidemiology and aetiological factors. There is brief mention of tumour immunology and the problem of immunosuppression. The second section deals with the management of patients with cancer. Treatment methods including the role of surgery, radiotherapy, chemotherapy and hormone therapy are dealt with in general terms, and provide a good introduction for the student. An important final part of the book is concerned with the multidisciplinary approach, techniques for early diagnosis, terminal care and public education.

This is not a treatise designed as a reference for the therapy of specific cancers; several books of this type are available. This book fulfills a different need, and should provide both undergraduates and laboratory scientific staff with a useful introduction to modern cancer treatment. The text is well laid out and easy to read. I can recommend this book for undergraduate teaching. It is not too long and is one of the few books which provides a good basic scientific introduction together with the modern approach to patient management.

D. CROWTHER